# Antimicrobial Peptides can Enhance the Risk of Persistent Infections

**DOI:** 10.3389/fimmu.2012.00222

**Published:** 2012-08-01

**Authors:** Marina Berditsch, Sergii Afonin, Tatiana Vladimirova, Parvesh Wadhwani, Anne S. Ulrich

**Affiliations:** ^1^Institute of Organic Chemistry, Karlsruhe Institute of TechnologyKarlsruhe, Germany; ^2^Institute of Biological Interfaces-2, Karlsruhe Institute of TechnologyKarlsruhe, Germany

Bacterial resistance against conventional antibiotics is an escalating problem in modern medicinal treatment of infectious diseases, as a growing number of immunocompromised patients are suffering from hospital-acquired bacterial colonization. The spreading of genetic bacterial resistance has stimulated the interest in natural antimicrobial peptides (AMPs) as promising drugs against pathogenic species (Giuliani et al., 2007; Zaiou, 2007; Fjell et al., 2012). These peptides are a fundamental component of the innate immune system and represent the first line of defense against a broad spectrum of microorganisms (Radek and Gallo, 2007). Owing to their intrinsic amphiphilic character and usually positive net charge, AMPs bind strongly to bacterial plasma membranes. These receptor-independent interactions, which are present already at sub-lethal concentrations, can induce lipid flip-flop, cause the formation of stable or transient pores, and/or lead to membrane depolarization and permeabilization (Spindler et al., 2011). Elevated AMP concentrations tend to fully disrupt the lipid bilayer barrier, thereby exhibiting an immediate detergent-like action that kills the cells. In the latter scenario, it is impossible for the bacteria to develop any resistance or tolerance against AMPs. In addition to these primary effects on the plasma membrane, several AMPs were also shown to translocate across the lipid bilayer and interact with various intracellular targets. Such alternative targets include membrane respiratory proteins, nucleic acids, as well as machineries of cell wall and protein biosynthesis (Epand and Vogel, 1999; Zhang et al., 2001; Brogden, 2005; Giuliani et al., 2007; Mogi and Kita, 2009; Spindler et al., 2011).

Unlike conventional antibiotics, AMPs do not tend to operate in a stereospecific manner. Nevertheless, some resistance mechanisms have evolved, such as a reduction of the net negative charge in the bacterial envelope, the active efflux removal of AMPs, or their proteolytic destruction (Peschel, 2002; Otto, 2009). However, most pathogenic Gram-positive and Gram-negative bacteria still remain susceptible to AMPs, and much promise is associated with their further development and application. Currently, the high production costs of natural mammalian AMPs and their low molecular stability are considered to be a drawback for reaching the drug market (Zaiou, 2007). Here, we demonstrate that another, as yet unknown problem may emerge in the use of AMPs. Namely, some AMPs can cause bacterial persistence, a phenomenon known to be associated with the formation of biofilms responsible for chronic diseases (Lewis, 2010). These biofilms, in turn, have a high tolerance against conventional antibiotics and represent a dangerous growth form of pathogenic bacteria that should be avoided by all means (Stewart and Costerton, 2001).

Amongst the best-studied AMPs are Magainin-2 (Mag2) and PGLa from *X. laevis*, commonly both denominated as “magainins”. These cationic peptides are produced in the granular glands of the frog skin and acquire an amphiphilic α-helical structure when bound to lipid membranes. Both peptides are moderately active *per se*, but a notable feature is their synergistically enhanced action in a 1:1 mixture (Matsuzaki et al., 1998; Strandberg et al., 2009). Magainins were used as a blueprint to design an ideal α-helical “model amphiphilic peptide” MAP (Oehlke et al., 1998), which also exhibits an antibacterial effect (Palm et al., 2006). All three helical molecules (with a length of around 20 amino acids) bind to lipid bilayers, where they can be surface-bound or obliquely immersed, depending on peptide concentration (Glaser et al., 2005; Bürck et al., 2008; Strandberg et al., 2009) and temperature (Afonin et al., 2008b). A fully inserted transmembrane state of these peptides has been associated with the formation of transient pores (Afonin et al., 2008b; Ieronimo et al., 2010). These have been shown to allow the escape of small metabolites and ions (Matsuzaki, 1998), thereby also decreasing the transmembrane proton gradient and as a result ATP generation. Mag2 was found to enhance the uncoupling and depolarizing activity of PGLa in liposomes containing cytochrome oxidase (Westerhoff et al., 1995). However, it is important to note that pore formation *per se* does not result in complete lysis of the membrane, and it does not kill bacteria (Epand and Vogel, 1999; Zhang et al., 2001). Here, we demonstrate that amphiphilic AMPs can actually stimulate survival mechanisms in bacteria instead of eradicating them.

For many pathogenic bacteria is known that under stress conditions (e.g., high bacterial density, depletion of nutrients and oxygen, temperature shift, osmotic shock, or selective pressure of antibiotics), cell populations produce small and temporary subpopulations of dormant cells. They are called persisters, because they have adapted to a long-term survival by a reduced level of metabolic activity, diminished protein synthesis, multidrug tolerance to antibiotics, and an enhanced ability to grow as surface-adherent biofilms. This general survival strategy is explained by an expression of starvation-related (Fux et al., 2005) or persister genes (Lewis, 2010). Due to their reduced growth rate, these reversible bacterial states are known as non-growing and “viable but non-culturable” (Oliver, 2005), or as slow growing phenotypes. The latter ones manifest on agar media as small colony variants (SCVs; von Eiff et al., 2006; Wellinghausen et al., 2009). Clinical isolates of *S. aureus* SCVs are characterized by deficiencies in transmembrane electron transport and ATP generation, and by an increased expression of adhesins instead of other virulence factors. These metabolic alterations facilitate the protective internalization of the bacteria into host cells and thereby persistent and recurrent infections (Proctor et al., 2006). The first identified molecular mechanism of a cellular stress response, which has been recently described in *E. coli*, explains how cells can convert into a dormant state. In the presence of stress factors, such as ciprofloxacin, which inhibits biosynthesis of DNA, the cells express the small autotoxic protein TisB. Interaction of this 29 amino acid peptide with the plasma membrane decreases the protonmotive force, reduces ATP synthesis, and thereby transforms bacteria into an isogenic dormant state (Dorr et al., 2010). Notably, in both modes of stress response (i.e., in slow growing auxotrophic clinical isolates of *S. aureus*, as well as in *E. coli* expressing TisB) the key feature is the disturbance of the proton gradient. Either by a deficiency in its generation or by its active dissipation, the reduced protonmotive force leads to a lower level of ATP generation by oxidative phosphorylation. The need for ATP production can be compensated by substrate-level phosphorylation, e.g., via glycolysis or via the arginine deiminase pathway. This general metabolic switch is associated with the characteristic slow growth in the biofilm mode (Proctor et al., 2006).

**Figure 1:**
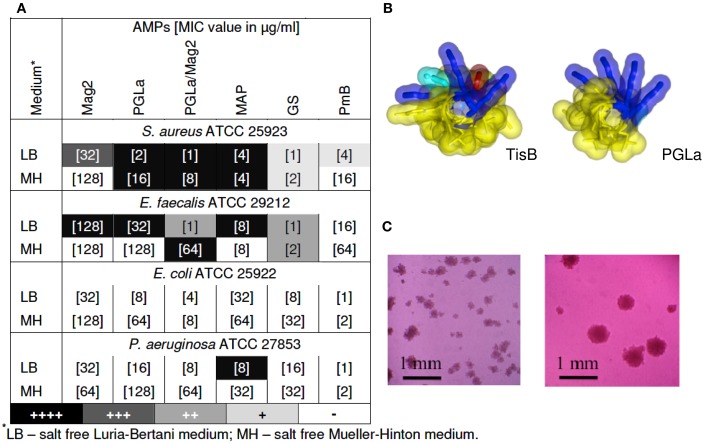
**(A)** Emergence of small colonies at sub-MIC concentrations of AMPs. MIC values are indicated in square brackets, as determined after 24 of exposure to the AMP by means of a resazurin color change. Pinpoint colonies were detected in the wells with concentrations corresponding to 1/2 MIC for all peptides, except for MAP, which was effective even at 1/4 and 1/8 of MIC. Occurrence of pinpoint colonies was observed as an average of at least two independent experiments; **(B)** Molecular structures of TisB (left) and PGLa (right); **(C)** Pinpoint colonies of *S. aureus* in the presence of MAP were detected on the bottom of 96-wells microtiter plates with an inverted microscope (Leica DM IL) at 100-fold magnification – two individual microcultures under identical conditions are shown as examples.

Our key idea, which elicited the present study, was the realization that TisB has a remarkably similar amphiphilic α-helical structure when compared to the antimicrobial magainin peptides described above (see Figure 1B). After all, both types of peptides increase the proton permeability of lipid bilayers. The stress response peptide TisB has been shown to localize to the inner membrane of *E. coli* (Unoson and Wagner, 2008), where it gets inserted in a transmembrane alignment (Steinbrecher et al., in revision). We thus wanted to find out whether membrane-active AMPs would also be able to trigger the formation of persister cells via their known depolarizing effect on bacteria. We monitored bacterial growth using the redox indicator resazurin (alamarBlue^™^), which changes its color from blue to pink upon reduction, as an indicator of cellular respiration. In this way, we carried out twofold microdilution assays with several different AMPs to determine their minimum inhibitory concentration (MIC) values from the bacterial respiratory activities. Subsequently, the wells of the microtiter plates were inspected with a microscope to see whether pinpoint colonies or biofilms had formed. Besides the α-helical PGLa, Mag2, a 1:1 mixture of PGLa/Mag2, and MAP, we also included two cyclic AMPs in this study. Gramicidin S (GS) and polymyxin B (PmB) have a very different molecular structure compared to the magainins, and they are rather selective against either Gram-positive or Gram-negative strains, respectively. PmB has a high affinity to Lipid A and allows rapid detoxification of LPS, thus exerting an immediate anti-inflammatory effect. It also inhibits respiratory proteins directly, such as an alternative NADH dehydrogenase and malate-quinone oxidoreductase (Mogi and Kita, 2009). PmB has been shown to kill *P. aeruginosa* already at peptide concentrations below the level that is needed to cause cellular depolarization (Zhang et al., 2000). The antibacterial action of GS is even more multifaceted. In addition to non-lethal depolarization, presumably via formation of transient pores at sub-MIC concentrations (Zhang et al., 2001; Afonin et al., 2008a), and membrane disruption at supra-MIC (Hartmann et al., 2010), GS also inhibits membrane-associated proteins such as cytochrome *bd* quinol oxidase and an alternative NADH dehydrogenase (Mogi and Kita, 2009), as well as various membrane-bound ATPases (Zhao and Dhalla, 1991). In addition, all ATP-dependent processes should be indirectly affected, since cyclic peptide GS has a particularly high affinity to bind ATP molecules (Krauss and Chan, 1983).

As anticipated from our structural and functional comparison of TisB with AMPs, we indeed found that tested bacteria formed small colonies upon treatment with AMPs. The gray scale of the shading in the table of Figure 1A summarizes the observed occurrence of adherent colonies in the liquid microcultures of various Gram-positive and Gram-negative strains. Represented here are the findings at sub-lethal concentrations of the different AMPs, usually taken at 1/2 of the corresponding MIC value (the actual MIC values are listed in square brackets). TisB was used as a comparison and lead to intensive biofilm-like growth in both Gram-positive strains and in *E. coli* K12 (data not shown). At sub-MIC levels of the AMPs, it is clear that some cells will survive in the original 10^5^–10^6^ CFU/ml inoculation dosis of the planctonic microcultures. However, the formation of small adherent colonies in the liquid medium, as illustrated in Figure 1C, suggests that these surviving bacteria have changed their phenotype due to the exposure to AMPs. Remarkably, all of the tested AMPs showed this effect, though to a different extent. MAP was the most effective, leading to pinpoint colonies in *S. aureus*, *E. faecalis*, and *P. aeruginosa*. Besides a notable dependence on the cultivation medium, the helical peptides (PGLa, Mag2, PGLa/Mag2, MAP) were generally found to be more effective than the cyclic GS and PmB. Interestingly, for the synergistic pair Mag2/PGLa, the occurrence of SCVs exceeded the corresponding effect of the individual peptides, thereby demonstrating a pronounced synergy in the promotion of bacterial survival. Pinpoint colonies were not found for *E. coli* ATCC 25922, which suggests a species dependent variability in the bacterial response.

It is known that sub-lethal levels of conventional bactericidal agents can induce mutagenesis (Kohanski et al., 2010), but can also enhance bacterial adherence to the host cells (Zhanel and Nicolle, 1992) and biofilm formation (Kaplan, 2011). The induction of survival mechanisms upon incubation with membrane-depolarizing AMPs, as demonstrated here for the first time, shows that these mechanisms are universal and can lead to an emergence of tolerance which does not imply genetic resistance. It might be considered as a general mechanism of evolutionary significance that the passage of protons across the plasma membrane reduces metabolic activity at stress conditions and converts bacteria into dormancy. This way, bacterial genomes are preserved from extinction as a biological species. Our findings have shown that sub-lethal AMP concentrations, at which there is no membranolytic effect, can trigger the transition of bacteria into dormancy, so that they can survive and grow in an adherent form even in liquid media. An alternative possibility to explain the emergence of the small pinpoint colonies could be the inability of the AMPs to kill existing dormant cells that might have been present in the inoculum. Both possibilities require further studies of the phenomenon. Independent of the mechanism, we can conclude that exposure to sub-lethal concentrations of AMPs, especially in the case of amphiphilic α-helical peptides, can enhance the risk of persistent bacterial infections.

Our findings do not imply, however, that AMPs cannot be used as antibacterial drugs in general. Since different types of peptides show different modes of membrane perturbation (c.f. cyclic GS and PmB), they are likely to bear different risks of triggering persistence and/or biofilm formation. Furthermore, higher organisms have evolutionarily optimized their strategies to cope with the universal bacterial stress response. For example, in the case of injury, the immune cells that express high levels of defensins and cathelicidins are attracted directly to the wound. This way, the AMPs are delivered at very high local concentration to the site of potential infection (Radek and Gallo, 2007; Zaiou, 2007; Hancock et al., 2012). Analogous local or topical applications of AMPs have helped and will continue to help the human organism, while avoiding an unfavorable imbalance in the usual, e.g., intestinal microbiota. We believe that medical approaches show most promise, when they can provide high local concentrations of AMPs directly at the site of infection. For instance, cationic AMPs can be delivered into the respiratory tract with an ultrasonic nebulizer (Falagas et al., 2010), and tissue-specific local drug delivery is possible via medical electrophoresis or using polymeric nanosphere gels (Batheja et al., 2011).
